# Subclinical Depressive Symptoms and Continued Cannabis Use: Predictors of Negative Outcomes in First Episode Psychosis

**DOI:** 10.1371/journal.pone.0123707

**Published:** 2015-04-15

**Authors:** Itxaso González-Ortega, Susana Alberich, Enrique Echeburúa, Felipe Aizpuru, Eduardo Millán, Eduard Vieta, Carlos Matute, Ana González-Pinto

**Affiliations:** 1 Department of Psychiatry, University Hospital of Alava-Santiago, University of the Basque Country, CIBERSAM, Vitoria, Spain; 2 Araba Research Unit, Osakidetza, Vitoria, Spain; 3 Dirección de Asistencia Sanitaria, Osakidetza, Vitoria, Spain; 4 Bipolar Disorders Program, Institute of Neurosciences, Hospital Clinic, University of Barcelona, IDIBAPS, CIBERSAM, Barcelona, Spain; 5 Achucarro Basque Center for Neuroscience, University of the Basque Country, CIBERNED, Leioa, Spain; Maastricht University, NETHERLANDS

## Abstract

**Background:**

Although depressive symptoms in first episode psychosis have been associated with cannabis abuse, their influence on the long-term functional course of FEP patients who abuse cannabis is unknown. The aims of the study were to examine the influence of subclinical depressive symptoms on the long-term outcome in first episode-psychosis patients who were cannabis users and to assess the influence of these subclinical depressive symptoms on the ability to quit cannabis use.

**Methods:**

64 FEP patients who were cannabis users at baseline were followed-up for 5 years. Two groups were defined: *(a)* patients with subclinical depressive symptoms at least once during follow-up (DPG), and *(b)* patients without subclinical depressive symptoms during follow-up (NDPG). Psychotic symptoms were measured using the Positive and Negative Syndrome Scale (PANSS), depressive symptoms using the Hamilton Depression Rating Scale (HDRS)-17, and psychosocial functioning was assessed using the Global Assessment of Functioning (GAF). A linear mixed-effects model was used to analyze the combined influence of cannabis use and subclinical depressive symptomatology on the clinical outcome.

**Results:**

Subclinical depressive symptoms were associated with continued abuse of cannabis during follow-up (β= 4.45; 95% confidence interval [CI]: 1.78 to 11.17; *P* = .001) and with worse functioning (β = -5.50; 95% CI: -9.02 to -0.33; *P* = .009).

**Conclusions:**

Subclinical depressive symptoms and continued cannabis abuse during follow-up could be predictors of negative outcomes in FEP patients.

## Introduction

Substance use disorders are commonly associated with other comorbid psychiatric disorders [1], [2]. Specifically, cannabis use has been associated with mental health problems [3], [4], [5], increased risk of psychosis [5], [6], [7], [8] and earlier onset of psychosis [9],[10],[11]. Once psychosis has begun, continued cannabis use has been associated with poorer functional outcomes [12],[13],[14]. Recent intervention studies aimed at reducing cannabis use and improving the prognosis of patients with recent-onset psychosis have been unsuccessful with low rates of effectiveness [15], [16]. Consequently, researchers have focused on identifying factors that mediate continued use of cannabis and some studies indicate that depressive symptoms may be a relevant factor [17],[18],[19].

Depressive symptoms of all levels of severity are common in patients with first-episode psychosis (FEP) [20], [21], [22], [23], [24], irrespective of whether they are cannabis abusers or not [16], [19]. Studies examining depressive symptoms in FEP patients (with and without cannabis abuse) have found that those with depressive symptoms have poorer outcomes with more suicidal thinking, more negative symptoms and worse functionality during follow-up [23], [24]. In a recent systematic review, anxiety and depression in FEP patients were related to the severity of psychotic symptoms and to worse clinical outcomes [19].

The evidence for an association between cannabis abuse and the type or number of depressive symptoms is conflicting. According to the self-medication hypothesis, subclinical depressive symptoms can cause social difficulties and psychological distress that may be relieved by cannabis abuse, [25]. Some studies have found an association between cannabis abuse and the presence of fewer or less severe depressive symptoms [26], [27], suggesting that cannabis use may alleviate some of these symptoms. Other studies, however, have observed an increase in negative [28] or depressive [17], [29] symptoms when Δ9-tetrahydrocannabinol (THC), the main psychoactive constituent of cannabis, is administered to patients with schizophrenia. Therefore, it remains unclear whether cannabis abuse relieves or induces depression-related symptoms. In a previous longitudinal study, FEP patients who stopped using cannabis had improved functioning and a significant reduction in negative symptoms in the long-term, whereas continued cannabis use was associated with poorer long-term outcomes [14]. From a neurobiological perspective, it is well known that THC acts on the cannabinoid system in the brain, which plays a role in emotional regulation [30]. However, the relationship between cannabis use and depressive symptoms is complex. Recent findings suggest the effect of cannabis use on depressive symptoms might be moderated by genetic factors, specifically the short allele of the serotonin transporter gene-linked polymorphic region (5-HTTLPR) genotype; i.e., cannabis use increases the risk for a worsening of depressive symptoms in individuals with this genetic vulnerability [30].

Thus, there is a strong evidence for the negative impact of prolonged cannabis use on long-term clinical and functional outcomes in FEP patients [13], [14], [31]. However, little is known about the influence of depressive symptoms on the long-term functioning of FEP patients with cannabis abuse, or the effect of depressive symptoms on the ability of patients to stop cannabis consumption [17], [18], [19]. Specifically, subclinical depression (i.e., a level of depressive symptoms that are below the threshold for a diagnosis of clinical depression), which is more common in these patients during follow-up, has not been studied.

The aims of this study were to examine the influence of subclinical depressive symptoms on long-term functional and clinical outcomes, and on the ability of patients to stop using cannabis. We hypothesized that patients with FEP who use cannabis and have persistent subclinical depressive symptoms during follow-up would have poorer functional and clinical outcomes, as well as a worse course for their addictive disorder, compared with patients without depressive symptoms during follow-up.

## Material and Methods

### Subjects

The study was conducted on patients who were cannabis users admitted for the first time with a first psychotic episode to Alava University Hospital of Alava-Santiago between 2003 and 2007. A first psychotic episode was defined according to the revised fourth edition of the *Diagnostic and Statistical Manual of Mental Disorders* (DSM-IV-TR) [32]. Cannabis use/abuse/dependence was defined according to DSM-IV-TR criteria and the scores of the European adaptation of the fifth version of the Addiction Severity Index (Europ-ASI) [33] (Table 1), and from information obtained from urine drug analyses. The FEP patients included in the study were aged 16–45 years and met the DSM-IV-TR diagnostic criteria for one of the following disorders: schizophreniform disorder; schizoaffective disorder; schizophrenia; delusional disorder; brief psychotic disorder; atypical psychosis; bipolar I/II disorder. Patients with major depression, substance-induced psychotic disorders, mental retardation or organic brain disorders were excluded from the study.

**Table 1:** Severity of Cannabis Use.

Severity of consumption	DSM-IV-TR^a^ criteria for abuse or dependence	Europ-ASI^b^ scores
Dependence	Meet minimal or more DSM-IV criteria for cannabis dependence	8–9
Abuse	Meet ≥1 criteria for cannabis abuse	4–7
Use	Abuse criteria but do not meet temporal criteria (at least 12 mo) or use 12 mo but not fulfilling any criteria of DSM-IV abuse	2–3
No use	No significant symptoms	0–1

^a^Fourth edition of the Diagnostic and Statistical Manual of Mental Disorders, revised edition (DSM-IV-TR).^23^

^b^European Addiction Severity Index (Europ-ASI).^25^

### Procedure

Patients were assessed clinically by trained psychologists or psychiatrists at baseline and at 1, 3, and 5 years of follow-up. The baseline assessment was conducted during the hospitalization for the first episode psychotic. Urine drug screens were performed at the follow-up assessments and a positive result confirmed that cannabis or other substances were being used. The study was approved by the Ethics Committee (Institutional Research Board) of University Hospital of Alava-Santiago. All participating patients were enrolled after providing written informed consent. In case of patients not capacitated to consent or minor patients, a legally authorized representative consented on the behalf of participants.

### Assessments

The DSM-IV-TR axis I diagnosis was made using the Structured Clinical Interview for DSM-IV-TR (SCID-I) [34]. Psychotic symptoms were measured using the Positive and Negative Syndrome Scale (PANSS) [35], [36], depressive symptoms were measured using the Hamilton Depression Rating Scale (HDRS-17) [37], [38], and psychosocial functioning was assessed using the Global Assessment of Functioning (GAF) [39], [40]. The clinical interview was carried out independently by two experienced clinicians to ensure the reliability of diagnoses. The data on inter-rater reliability obtained were satisfactory for SCID-I diagnoses (κ = 0.86) and for the scales used: PANSS (κ = 0.78); HDRS-17 (κ = 0.79); GAF (κ = 0.93) and Europ-ASI (κ = 0.90).

Severity of cannabis use was determined according to the DSM-IV-TR criteria for abuse or dependence and the 9-point scale of the Europ-ASI [33], [41] and patients were categorized as: no use, use, abuse and dependence as shown in Table 1.

For determining the influence of subclinical depressive symptoms on clinical outcomes and their potential relationship to quitting cannabis use during follow-up, patients were classified into 2 groups according to the presence of subclinical depressive symptoms during follow-up: (1) patients with subclinical depressive symptoms at least once during follow-up (DPG); and (2) patients without subclinical depressive symptoms during follow-up (NDPG). Subclinical depressive symptoms (i.e., depressive symptoms below the threshold for a diagnosis of clinical depression) were defined as present when the HDRS-17 score was greater than or equal to 8 (score range is 0–54), [42], [43].

### Data Analysis

Analyses were carried out using the Statistical Package for the Social Sciences (SPSS) version 21.0 for Windows and R 2.5.1 [44]. Descriptive summary statistics (means, standard deviations [SD], frequencies, percentages) were used to describe the sociodemographic and baseline clinical characteristics of the total patient sample and by the presence/absence of subclinical depressive symptoms during follow-up. Differences at baseline between the two depressive symptom groups (DPG and NDPG) were performed using the χ^2^ test or Fisher test for categorical variables, and the Student *t*-test or Mann-Whitney *U* test for continuous variables, depending on whether normality and size assumptions held. The variables with significant differences between groups were used as covariates in the subsequent analyses, after checking the collinearity.

A linear mixed-effects model was used to analyze the combined influence of cannabis use and subclinical depressive symptomatology on the clinical outcome of patients. The dependent variable was the GAF score. Cannabis use and depressive symptoms (HDRS-17 score ≥8) were included as fixed-effects and patient was included as a random effect in the model. Assessments at 1, 3 and 5 years after the first episode of psychosis were accounted for in the analysis. The maximum likelihood test was used to assess goodness of fit. Analysis of variance (ANOVA) was used to assess inclusion in the final model of the depression and cannabis use interaction term. Results are expressed as β coefficients with 95% confidence intervals (CI) and *P* values.

Logistic regression models were used to analyze the association between the subclinical depressive symptoms during follow-up (present or absent) and clinical outcomes at 5 years. Data are presented as odds ratios (OR) with 95% CIs and *P* values. Differences between groups (DPG and NDPG) in the severity of the other substance consumption (alcohol, tobacco, etc) were analyzed using Fisher exact tests.

## Results

### Baseline Characteristics of the Sample

At baseline, 74 patients met the inclusion criteria and were enrolled in the study. However, 6 patients dropped out of the study during follow-up and a further 4 patients were excluded from the analysis because they had a diagnosis of major depression during follow-up. Thus, the total sample analyzed was 64 FEP patients with cannabis use who were followed-up for 5 years. At baseline, 17 (26.6%) patients had bipolar disorder, 16 (25%) had schizophrenia, and 31 (48.4%) had other psychotic disorders (13 patients had brief psychotic disorder and 18 patients had psychotic disorder not otherwise specified). The sociodemographic and baseline clinical characteristics of the 10 patients excluded from the analysis did not differ from those of the patients included the analysis.

Of the 64 patients in the total sample, 36 (56.3%) were in the DPG group and 28 (43.8%) were in the NDPG. Table 2 summarizes the sociodemographic and baseline clinical characteristics of the total sample and of the two groups by presence/absence of subclinical depressive symptoms during follow-up (DPG, NDPG). These two groups did not differ, except for socioeconomic status and level of education (Fisher, *P* <. 05); DPG patients had a lower socioeconomic status and lower level of education. The mean HDRS-17 score did not differ between the DPG and NDPG groups. The severity of cannabis use did not differ significantly between groups (Fisher, *P* = .054). In the DPG group, 8.3% of patients met the criteria for cannabis use and 91.7% for cannabis abuse. In the NDPG group, 10.7% of patients met the criteria for cannabis use, 75% for abuse, and 14.3% for dependence. There were not significant differences between groups in alcohol, tobacco and other substances (cocaine, amphetamine, and heroine).

**Table 2:** Sociodemographic and Baseline Clinical Characteristics of the Total Sample and by Subclinical Depressive Symptomatology During Follow-up.

		Total (*n* = 64)	DPG^a^ (*n* = 36)	NDPG^b^ (*n* = 28)	*P* Value
Sex	Male	37 (57.8%)	17 (47.2%)	20 (71.4%)	χ^2^ = 3.78 (*P* = .052)
	Female	27 (42.2%)	19 (52.8%)	8 (28.6%)	
Age		24.75 ± 5.99	24.58 ± 4.93	24.96 ± 7.21	U = 485.5 (*P* = .802)
Civil status	Single	57 (89.1%)	31 (86.1%)	26 (92.9%)	Fisher (*P* = .840)
	Married	4 (6.3%)	3 (8.3%)	1 (3.6%)	
	Other	3 (4.7%)	2 (5.6%)	1 (3.6%)	
Socioeconomic status	Low	17 (26.6%)	14 (38.9%)	3 (10.7%)	Fisher (***P* = .004**)
	Medium	41 (64.1%)	19 (52.8%)	22 (78.6%)	
	High	6 (9.4%)	3 (8.3%)	3 (10.7%)	
Education level	No education	3 (4.8%)	2 (5.6%)	1 (3.7%)	Fisher (***P* = .044**)
	Primary school	21 (33.3%)	12 (33.3%)	9 (33.3%)	
	Secondary school	34 (54%)	22 (61.1%)	12 (44.4%)	
	College	5 (7.9%)	0 (0%)	5 (18.5%)	
Occupation	Active	28 (43.8%)	17 (47.2%)	11 (39.3%)	Fisher (*P* = .125)
	Unemployed	19 (29.7%)	13 (36.1%)	6 (21.4%)	
	Other	17 (26.5%)	6 (16.7%)	11 (39.3%)	
Cannabis use	Use	6 (9.4%)	3 (8.3%)	3 (10.7%)	Fisher (*P* = .054)
	Abuse	54 (84.4%)	33 (91.7%)	21 (75%)	
	Dependence	4 (6.3%)	0 (0%)	4 (14.3%)	
Other substances	Tobacco	54 (84.4%)	29 (80.6%)	25 (89.3%)	χ^2^ = 0.64 (*P* = .425)
	Alcohol	42 (65.6%)	26 (72.2%)	16 (57.1%)	χ^2^ = 1.59 (*P* = .208)
	Other	34 (53.1%)	17 (47.2%)	17 (60.7%)	χ^2^ = 1.15 (*P* = .283)
Antipsychotics	Atypical	44 (68.8%)	29 (80.6%)	15 (53.6%)	χ^2^ = 3.36 (*P* = .067)
	Typical	20 (31.3%)	12 (33.3%)	8 (28.6%)	χ^2^ = 0.15 (*P* = .698)
PANSS	Positive	25.47 ± 6.44	25.19 ± 7.34	25.82 ± 5.16	U = 491 (*P* = .860)
	Negative	17.48 ± 8.76	18.08 ± 8.77	16.71 ± 8.84	U = 452 (*P* = .481)
	General	41.56 ± 10.38	41.67 ± 8.97	41.43 ± 12.21	U = 451 (*P* = .473)
GAF		50.48 ± 15.22	49.78 ± 12.11	51.39 ± 18.67	U = 489 (*P* = .839)
HDRS-17		18.55 ± 7.20	17.44 ± 8.17	19.96 ± 5.54	U = 417.5 (*P* = .241)

*Notes*: Data are presented as mean ± SD or *n* (%). The percentage given for each variable refers to the total *n* available for that variable. P values are results of Chi-square (categorical variables) and Mann-Whitney *U* tests (continuous variables). Values in bold are significant at *P*<.05. PANSS, Positive and Negative Syndrome Scale; GAF, Global Assessment of Functioning; HDRS-17, Hamilton Depression Rating Scale.

^a^DPG = patients with subclinical depressive symptoms at least once during the 5 year follow-up.

^b^NDPG = patients without subclinical depressive symptoms during the 5 year follow-up.

### Follow-up Results

#### Association between Severity of Cannabis Use, Depressive Symptoms and Functioning

The results of the fixed-effects model showed that the presence of subclinical depressive symptoms during follow-up (HDRS-17 score ≥ 8) was associated with continued cannabis abuse (4–7 points on the Europ-ASI scale) during follow-up (β = 4.45; 95% CI: 1.78 to 11.17; *P* = .001) and with worse functioning during follow-up (β = -5.50; 95% CI: -9.02 to -0.33; *P* = .009) (1, 3 and 5 years). In addition, cannabis abuse during follow-up was associated with a lower score on the GAF scale (i.e. poorer functioning), compared with no use of cannabis during follow-up (β = -4.71; 95% CI: -9.04 to -0.31; *P* = .038), whereas cannabis use during follow-up (2–3 points on the Europ-ASI scale) was not significantly associated with functioning (β = -2.03; 95% CI: -11.40 to 7.34; *P* = .669). The effects of depressive symptoms and cannabis use were independent because the interaction term for these two variables showed no significant influence on the GAF score (*P* = .863).

#### Other Outcomes at Follow-up

The clinical status of patients at each of the follow-up evaluations is provided in Table 3. Logistic regression analyses of the association between depressive symptoms and clinical symptomatology at 5-years follow-up showed that the presence of subclinical depressive symptoms during follow-up (DPG) was associated with more PANSS positive symptoms (OR = 1.20; 95% CI: 1.07 to 1.36; *P* = .004), PANSS negative symptoms (OR = 1.13; 95% CI: 1.05 to1.23; *P* = .003), and PANSS general symptoms (OR = 1.21; 95% CI: 1.10 to 1.33; *P* <. 001) at year 5 than the absence of subclinical depressive symptoms during follow-up (NDPG). DPG was also associated with poorer psychosocial functioning (OR = 0.92; 95% CI: 0.88 to 0.96; *P* <. 001) compared with NDPG. In addition, the clinical course was worse for patients in the DPG group; they had more than twice the probability of having hospitalizations (OR = 2.07; 95% CI: 1.29 to 3.30; *P* = .002) and episodes of psychosis (OR = 2.55; 95% CI: 1.51 to 4.31; *P* <. 001) by year 5 of follow-up, compared with the NDPG group. The mean number of hospitalizations during the 5-year follow-up was 4.41 (SD 3.29) and 1.89 (SD 1.23) in the DPG and NDPG groups, respectively. Likewise, the mean number of psychotic episodes was 5.03 (SD 2.93) vs 2.29 (SD1.21) in the DPG and NDPG groups, respectively. The mean (SD) number of suicide attempts did not differ between the DPG (0.38 ± 0.78) and NDPG groups (0.14 ± 0.36; *P* = .157).

**Table 3:** Clinical status of patients at follow-up.

		Total (*n* = 64)	DPG^a^ (*n* = 36)	NDPG^b^ (*n* = 28)
PANSS Positive	1 year	12.90±7.22	16.00±7.97	9.04±3.45
	3 years	13.25±6.59	16.24±6.88	9.71±4.05
	5 years	13.03±7.13	16.15±7.95	9.48±3.74
PANSS Negative	1 year	12.19±6.55	14.06±7.26	9.86±4.66
	3 years	14.28±8.14	17.42±8.94	10.57±5.12
	5 years	15.73±8.42	19.27±9.05	11.69±5.41
PANSS General	1 year	26.54±9.61	31.54±9.78	20.29±4.31
	3 years	29.69±11.12	33.61±11.39	22.53±6.01
	5 years	29.79±10.77	35.45±10.91	23.34±5.97
HDRS-17	1 year	8.87±7.13	12.00±7.44	4.96±4.32
	3 years	8.21±7.07	11.73±7.58	4.07±3.17
	5 years	8.32±5.88	11.82±5.23	4.34±3.64
GAF	1 year	61.94±14.63	57.60±14.37	67.36±13.26
	3 years	61.38±14.48	55.48±12.28	68.32±13.94
	5 years	63.00±16.14	55.21±14.93	71.86±12.65

*Notes*: Data are presented as mean ± SD. PANSS, Positive and Negative Syndrome Scale; GAF, Global Assessment of Functioning; HDRS-17, Hamilton Depression Rating Scale.

^a^DPG = patients with subclinical depressive symptoms at least once during the 5 year follow-up.

^b^NDPG = patients without subclinical depressive symptoms during the 5 year follow-up.

During the 5-years of follow-up, cannabis use was reduced in both the DPG and NDPG groups. There was no significant difference in the severity of cannabis use between the two groups at year 5 (Fisher, *P* = .056): 50% of the DPG group vs. 71.4% of the NDPG group did not use cannabis, 8.3% vs. 10.7% used cannabis, 41.7% vs. 14.3% abused cannabis, and 0% vs. 3.6% had cannabis dependence. Severity of alcohol use differed significantly between the DPG and NDPG groups at year 5 of follow-up (Fisher, *P* = .001): in the DPG group, 15.2% of patients did not use alcohol, 42.4% met the criteria for alcohol use, and 42.4% for alcohol abuse; while in the NDPG group, 12% of patients had no alcohol consumption, 80% were using alcohol, but only 4% were abusing or dependent on alcohol. Finally, the use of other substances did not differ between groups at year 5: 27.3% in the DPG group vs. 8% in the NDPG group; Fisher, *P* = .108.

## Discussion

This study has three main findings. First, FEP patients with cannabis abuse who have subclinical depressive symptoms during 5 years of follow-up have a poorer clinical and functional outcome, compared with patients without subclinical depressive symptoms during follow-up. Second, subclinical depressive symptoms during long-term follow-up are associated with continued cannabis abuse. Third, continued cannabis abuse is associated with poorer functional outcome.

Although other studies have examined long-term outcomes in FEP patients with regards to depressive symptoms [17], [18], [19], [23], [24], this is the first study to analyze the role of subclinical depressive symptoms in a sample of FEP patients who were cannabis users. Depressive symptoms often appear at the onset of early psychosis and subsequently decrease, remaining at a lower level during the course of the disease [20], [21], [22], [23], [24]. This persistence or presence of subclinical depressive symptoms is not universal, at least in patients with cannabis abuse. In our sample, we identified two groups of patients (those with and without subclinical depressive symptoms during follow-up) who clearly differed in their long-term outcomes. Patients with subclinical depressive symptoms during 5 years’ follow-up had poorer long-term outcomes, with more negative psychotic symptoms and poorer psychosocial functioning. These results are consistent with the study by Sönmez et al. [23] which showed that FEP patients with persistent depression had poorer functioning and more negative symptoms at 12 months follow-up. Birchwood et al. [45] have suggested that persistent depression is an essential aspect of schizophrenia that is associated with disease severity and negative symptoms. Our results show that the link between depression and negative symptoms is particularly important in patients with comorbid cannabis use, although the etiology of the association remains unclear [19]. There are at least three hypotheses that could explain the relationship between subclinical depressive symptoms and cannabis abuse: (1) depressive symptoms are a consequence of cannabis abuse [46]; (2) the self-medication theory [25], [47], [48], [49]; and (3) a common genetic or environmental vulnerability for both disorders [46]. It is possible that all three may play a role, although our results can only be discussed with regards to the first two hypotheses. Lynskey et al. [46] showed that patients with depressive episodes in adolescence were more likely to have subsequent cannabis dependence, and that cannabis dependence was associated with a higher risk of subsequent major depression in dizygotic, but not in monozygotic twins. Our results indicate that subclinical depressive symptoms are enough to make it difficult for FEP patients to stop using cannabis.

For the second hypothesis (self-medication), symptoms of apathy or lack of motivation facilitate the appearance of a depressed mood, which, in turn, is associated with an increased risk of cannabis consumption in an attempt by patients to relieve their negative emotional state [25]. Often these patients show a poor repertoire of behaviors, with a lack of rewarding activities in their daily life, except for the addictive substance [47]. This raises the question of whether cannabis use could be the consequence of subclinical depressive symptoms. Our results suggest that patients who have greater vulnerability to maintain subclinical depressive symptoms during follow-up are more likely to continue cannabis use in the long term, while patients without subclinical depressive symptoms are more able to discontinue cannabis consumption. One factor that may contribute to the difficulty in stopping cannabis use among patients with subclinical depression is the anxiolytic effects of its two main constituents, THC and cannabidiol [50], [51].

The third finding of this study is that FEP cannabis users who stopped using cannabis during follow-up had better functional outcomes. It is important to understand why some patients stopped using cannabis while others continued to use it. One possible explanation for failing to stop cannabis use, according to our results, is the presence of subclinical depressive symptoms during follow-up. Interestingly, there was no significant interaction between the independent variables cannabis use and depressive symptoms on functional outcome. This means that both cannabis abuse (β = -4.7), and depressive symptoms (β = -5.5) have a negative and independent effect on the functional outcome (GAF score), and when both are present the effect is additive rather than multiplicative. Because cannabis use and depressive symptoms have an independent effect on functioning, there must be a complex underlying mechanism.

Another finding of this study is that patients with subclinical depressive symptoms during follow-up not only had more positive psychotic symptoms in the long-term, but also more alcohol abuse. In a recent systematic review, Hartley et al. [19] found that the presence of anxiety and depressive symptoms is associated with severity of psychosis and with positive symptoms. Thus, depressive symptoms could be a potential target in the treatment of psychosis [19]. In a previous study, we observed that FEP patients who continued using cannabis during 8 years of follow-up exhibited poorer long-term functioning and had more negative symptoms than patients who stopped using cannabis [14].

This study has several limitations that must be taken into account when interpreting the findings. These limitations include the small sample size and the naturalistic setting of the study. The absence of a neuropsychological assessment is another limitation of the study, given that cognitive impairment has been reported to be a mediator of cannabis use and its relationship with depressive symptoms in some populations [52]. However, assessment of cognition was not a primary objective of the study. A further limitation is the low number of evaluations during the follow-up period, which does not allow us to determine any firm association between depression and poor functioning. The lack of premorbid assessments means that we cannot rule out that poor functioning was present before the start of cannabis abuse. In addition, at baseline the HDRS score is higher (although not significantly) in the NPDG group compared to the DPG group. These subtle differences at baseline would be difficult to observe under the conditions of severity of psychotic symptoms at admission for a psychotic episode. Finally, the role of sex should be considered in future studies, since depression is more common in females, but cannabis use is more prevalent in males.

The strengths of our study include the long follow-up for 5 years and the representativeness of the sample, which includes the majority of FEP patients who were cannabis users in psychiatric care in the geographic area of Vitoria.

## Conclusions

In conclusion, our findings suggest that subclinical depressive symptoms and continued cannabis abuse during long-term follow-up could be predictors of negative outcomes in FEP patients. Patients with a low level of depression are less likely to quit using cannabis and are at risk of experiencing more severe psychosis during follow-up. Subclinical depressive symptoms should be a therapeutic target in FEP patients to prevent the development of an unfavorable clinical and functional course, especially in cannabis users.
